# Reply to Editorial in February Number

**Published:** 1899-04

**Authors:** C. M. Wright

**Affiliations:** Cincinnati, Ohio


### REPLY TO EDITORIAL IN FEBRUARY NUMBER.

To the Editor:

Sir,—In the editorial pages of the February International Dental Journal a vigorous and scathing article appears against an anonymous correspondent of another journal, and an editor whose "moral sense was not above publishing a scurrilous letter." A selected part of the letter was quoted, and reference made to the December (1898) number of the *Dental Review* where the entire letter could be found. I had not seen the letter until attention was called to it by the doughty editorial in the International; then, after reading it with careful consideration, I really failed to discover the *offence* which could so excite the gush of gory ink from a critical editor's pen. In the first place, the brief quotations from the letter do not, it seems to me, justly express the trend of "Jesse's" criticisms, for the discussion of the physical and moral caliber of new students, and all such subjects, on the part of the chiefs of the Faculty Association were distinctly acknowledged as important, the following words of the letter expressing this: "*All of which is proper and just to everybody concerned.*" The ethical conceptions and business methods, the disreputable practices, or let us rather say, the unprofessional standards adopted by some of the colleges wearing the cloak of the Faculty Association in their methods of winning students, by employing runners, offering special inducements, "steering" of the unsuspecting, and making false promises,—seem to me to be the prominent and pertinent points in the criticisms of "Jesse" in the *Review* letter. It cannot be true that editors have rights of criticism that *nom de plume* writers have not.

A man may smile and smile and be a villain still, and a college may even teach anatomy and advertise "all the requirements of the Faculty Association strictly adhered to" in its catalogue, and yet resort to "skullduggery"—whatever that may imply—in its business methods.

If all the colleges which have been admitted to membership in the Association, and who wear the badge of the Association,—which, like the button of the Legion of Honor, should distinguish the wearer as high-toned in ethical as well as educational matters,—were pure in this respect, why is it borne in on the minds of teachers that students have, within a few years, imbibed the impression that *dollars* are an important factor as a force to influence deans and secretaries of first-class dental colleges? Why is it that the opinion prevails that dental colleges and large department stores are becoming more and more mercenary in character? The reference in the editorial to the great financial loss that the college sustains which was expelled from the Association shows the necessity for all sorts of ventures in the dental college business to acquire membership of the Faculty Association. This in a way may elevate the standard of dental education, but it does not necessarily make the college reputable as far as its business and ethical character is concerned.

From observations among practising and teaching *confrères,* I have to admit that these notions are held, and are becoming more general than they were, say, five years ago; and I am disposed to believe with "Jesse" that it has developed from the display of the competitive business spirit among colleges hiding under the mantle of the Faculty Association; therefore, when he points to this stain on the mantle (and I do not know of any better way for an outsider to do this than through the dental press), and calls upon the Association to notice it, and scour it till it shall be as Cæsar's wife, and wishes in his modesty to hide his name under a *nom de plume* (the name and rank must be known to the editor of the *Dental Review*), I fail to perceive the infamy of the thing, or the libellous character of the letter, or the guilt of the editor in publishing it.

I am rather disposed to regard the editorial as unfair and not quite *apropos,* and to beg of the writer of the same to review the matter and modify his "criticism."

Yours very truly,

C. M. Wright.

Cincinnati, Ohio.

